# Awareness of Pap testing and factors associated with intent to undergo Pap testing by level of sexual experience in unmarried university students in Korea: results from an online survey

**DOI:** 10.1186/1472-6874-14-100

**Published:** 2014-08-27

**Authors:** Hae Won Kim

**Affiliations:** 1 Research Institute of Nursing Science, College of Nursing, Seoul National University, Taehak-ro 103, Jongro-gu, Seoul, South Korea

**Keywords:** Cervical cancer, Pap test, University student

## Abstract

**Background:**

Young and unmarried women have not been a target group for cervical cancer prevention in Korea. No previous studies have investigated the awareness of Pap testing, the intention to undergo Pap testing, or the factors associated with that intention, in this group of women. This information would be useful for an expansion in the focus of primary cervical cancer prevention. This study aimed to compare the awareness of Pap testing between groups of unmarried university students in Korea, and to investigate the factors associated with the intention to undergo Pap testing, by level of sexual experience.

**Methods:**

A total of 475 unmarried university students who had never undergone a Pap test completed a web-based survey. Differences in awareness of the importance of the Pap test, confidence in Pap testing, intention to undergo the test, attitudes, subjective norms, perceived control, stigma, and shame by level of sexual experience were analysed using independent *t*-tests. Associations between measurement variables and intention to undergo Pap testing were analysed using correlation analysis. Variables yielding significant associations (*p* < 0.05) were included in a stepwise multiple regression model of intention to undergo Pap testing.

**Results:**

Most participants perceived that the need for regular Pap testing was less important (score, 77.76) than other methods of cervical cancer prevention. They were not confident that is was an effective method of cervical cancer prevention for themselves (score, 59.56). There were differences in confidence in Pap testing and in the factors associated with intention to undergo Pap testing between sexually experienced and sexually inexperienced students. Regardless of level of sexual experience, the subjective norm was the most important predictor of intention to undergo Pap testing.

**Conclusions:**

There was a low level of Pap screening awareness among the students. The factors associated with intention to undergo Pap testing differed by level of sexual experience. Social influence was an important factor that could be used to increase the intention to receive a Pap test in the university student population. Strategies to increase the intention to undergo Pap screening should be introduced and should be adapted to the level of sexual experience.

## Background

In 2010, cervical cancer was the seventh most prevalent type of cancer in Korean women, the third most prevalent cancer in Korean women aged 15–34 years, and the fifth most prevalent cancer in Korean women aged 35–64 years [1]. The age-standardised prevalence of cervical cancer in Korea appears to be gradually decreasing [2]. The Papanicolau (Pap) test is the most common cervical cancer screening test used in Korea and was adopted as a national health-screening method in 1988 [3]. The national health-screening program targets married women and women older than 30 years. Young and unmarried women have not been considered a target group for cervical cancer prevention. Little is known about their Pap screening behaviours.

Guidelines for early cervical cancer screening were published by the Korean Society of Obstetrics and Gynaecology and the National Cancer Centre in 2001. These guidelines recommend annual Pap testing for all women aged >20 years or after their first sexual intercourse, whichever occurs earlier [4]. However, the results of recent surveys revealed that only 4.8% of Korean university students had received a Pap test [5], and that only 5% of these students had been vaccinated against the human papilloma virus (HPV) [6]. In contrast, the uptake of Pap testing among their Western female university counterparts was almost 72%, and 68% of the women were first screened by the age of 18 years [7]. Thus, health professionals in Korea should make university students aware of the importance of Pap testing and encourage active participation in the Pap screening program for the early prevention of cervical cancer.

Evidence from many studies indicates that there is a vulnerability to cervical dysplasia and susceptibility to HPV infection among female university students [8-11]. This group of women has an insufficient understanding of Pap testing [12] and a lack of awareness regarding cervical cancer prevention in general [5,6,13]. Given that there is currently no mandatory HPV vaccine policy in Korea, young and unmarried women should be actively encouraged to undergo Pap screening [6]. No previous studies have investigated the awareness of Pap testing and the intention to undergo Pap testing, or the factors associated with that intention, in university students in Korea. This information is important for the guidance of relevant cervical cancer prevention strategies during the expansion of primary cervical cancer prevention programs. Thus, this study explored the antecedents or factors associated with intention to receive a Pap test and the degree of awareness of the importance of Pap testing and confidence in pap testing for cervical cancer prevention.

Regardless of cultural background, negative feelings such as stigma and shame are consistent obstacles to the decision to receive a Pap test [13-15]. The relationships between these feelings and the intention to undergo Pap testing were evaluated in the present study. The relationships between personal characteristics (e.g., age, religion, economic status, and sexual history) and the intention to undergo Pap testing were also examined [3,11,15,16].

Awareness, intentions, and factors related to Pap testing are influenced by a woman’s degree and type of sexual experience [6,13]. Because sexual experience can cause HPV infection and cervical dysplasia [9,10], it may also affect the perceptions of susceptibility to cervical cancer [17]. Whether the students were sexually active was a primary interest of this study, because students who have engaged in sex should begin Pap screening [4]. The differences in awareness of the Pap test, the intention to undergo Pap testing, and the factors associated with that intention could also depend on sexual experience, and were also examined.

The intentions and the attitudes toward Pap screening were assessed using the Theory of Planned Behaviour (TPB), which has been widely used in cervical cancer prevention research [7,13,18,19]. According to the TPB, a more favourable attitude will make a person more attentive toward a recommendation made by significant others (e.g., sexual partners, family members, and friends), and a greater perceived level of control will strengthen the intention to perform the behaviour in question [20]. The TPB framework was applied to Pap testing in this study, and it was postulated that such attitudes, subjective norms, and perceived control could be antecedents of the intention to undergo a Pap test. Future actual Pap screening behaviour may only be predicted from the intentions, especially among students who never experienced the Pap test. The significant antecedents of those intentions could then be applied to promote the Pap test.

The purpose of this study was to explore the awareness of cervical cancer prevention methods, including Pap testing, among unmarried female university students in Korea. Awareness of Pap testing, and intentions, attitudes, norms, and perceived control based on the TPB, stigma, shame, and sociodemographic factors were examined. Factors associated with the intention to undergo Pap testing, relative to sexual experience, were identified.

## Methods

### Subjects and data collection procedures

The research protocol was approved by the Myoung-ji University Hospital Institutional Review Board. Data were collected between November 15, 2012, and February 28, 2013, via a web-based survey. Subjects were included in the study if they currently attended one of 11 universities, were female and unmarried, and had not previously experienced a Pap test. Subjects were recruited using convenience sampling from several geographic regions via online advertisements. The subjects were accessed via the homepages of the 11 universities. These universities were selected from eight administrative regional clusters so as to ensure that the respondents proportionally represented the national geographic distribution of female university students in Korea. The students were informed of the survey via the “advertisement” page of the university homepages. Students who were willing to participate directly accessed the online survey webpage at http://research.kd.ac.kr/survey1. After accessing the survey, all subjects were anonymous and were coded into one of the eight regions. The survey began by requesting that the subject read and sign an informed consent form. A total of 480 female students completed the survey. Five respondents completed <50% of the survey’s sociodemographic items. Their responses were excluded from the analysis (n = 475).

### Measures

Sociodemographic data were the first data collected from each respondent. These data included each respondent’s age, religion, monthly allowance, whether she had previously heard of the Pap test, whether she was vaccinated against HPV, had a family history of cervical cancer, and information about her sexual history. Then, the awareness of Pap testing was assessed to avoid influencing the response to questions soliciting an opinion about the Pap test.

Each respondent indicated whether she knew when she should receive a Pap test by choosing one of following possible responses: “The Pap test is not necessary if I am HPV-vaccinated,” “I don’t know,” “The Pap test is necessary after marriage,” or “The Pap test is necessary before marriage.”

The following information was then conveyed to each respondent prior to asking her opinion regarding cervical cancer prevention (including Pap testing). This basic information was minimal, but it was essential so that all students could respond to the main survey: “The purpose of Pap test is to look for precancerous cell changes on the cervix that can be treated, so that cervical cancer is prevented. The Pap test can also detect cervical cancer early, when treatment is most effective [21]. According to the guidelines of the Korean Society of Obstetrics and Gynaecologic Oncology and Colposcopy, the Pap test is recommended annually for sexually active women aged older than 20 years [4]. The HPV vaccine is administered via intramuscular injection to prevent cervical cancer. The recommended age range for HPV vaccination is 9–26 years [22]”.

After giving this information, the perception of the importance and the confidence regarding the five cervical cancer prevention practices, including HPV vaccination, undergoing a regular Pap test, abstinence from sexual intercourse until marriage, minimising the numbers of sexual partners, and regular condom use according to the guideline for HPV and cervical cancer prevention [22,23], were individually assessed on a scale ranging from 0 (not at all) to 100 (very much).

The 18 TPB items related to the Pap test were developed based on Ajen’s theory [23]. The TPB variables and components (i.e., items) of each variable relevant to Pap screening were behavioural attitudes (five items), subjective norms (three items), perceived behavioural control (six items), and behavioural intention to undergo a Pap test (four items). All of the variables were assessed on a four-point scale ranging from 1 (not at all) to 4 (very much).

Higher subtotal scores indicated that respondents perceived greater beneficial effects or had a favourable attitude toward the Pap test. Higher scores also indicated that the respondent listened attentively to significant others, perceived that there were barriers in Pap test, and had greater intentions to undergo a Pap test.

Prior to survey analysis, the content of each item was validated by three experts on a four-point scale ranging from 1 (not at all) to 4 (very much). The results of this validation phase revealed that 16 of the 18 items were scored as very necessary (i.e., a score of 4), and two items were considered to be necessary (i.e., a score of 3). Therefore, all 18 items were factor-analysed for construct validation.

The reliabilities of the scales used for behavioural attitudes, subjective norms, perceived behavioural control, and behavioural intention to undergo a Pap test variables were confirmed by Cronbach’s α values of 0.86, 0.70, 0.68, and 0.90, respectively (total Cronbach’s α = 0.77). Prior to factor analysis, normal distributions were confirmed by random spreading from normal plots and detrended normal plots. The adequacies of the factor analysis were then identified using the Kaiser–Meyer–Olkin (KMO) measure (=0.82), and Bartlett’s test of sphericity (*χ*^2^ = 3100.93, *p* < 0.001). The principal axis rotation extraction with the oblimin rotation technique was used for the factor analysis to allow for determination of the inherent dimensions among the correlated variables [6,24]. The results indicated that the four factors of behavioural attitudes, subjective norms, perceived behavioural control, and behavioural intention to undergo a Pap test accounted for 58.30% of the variance, and explained 27.14%, 12.62%, 11.56%, and 6.98%, of the variance, respectively (Table 1).

**Table 1:** Validity and reliability: Theory of Planned Behaviour variables related to Pap testing, and stigma and shame variables

**Factor/items**	**Factor loading**	**Eigen- value**	**Variance (%)**	**Cronbach’s α coefficient**
Behavioural attitudes				
1) Pap testing is helpful for early detection of cervical cancer	0.73	4.89	27.14	0.86
2) Pap testing is effective for curing cervical cancer	0.68			
3) Pap testing is helpful for health management	0.75			
4) Pap testing makes me confident about my health status	0.65			
5) Pap testing is reliable for diagnosing cervical cancer	0.60			
Subjective norms				
1) Family and friends think Pap testing is necessary to detect cervical cancer	-0.46	2.27	12.62	0.70
2) Family and friends think Pap testing is necessary to detect cervical cancer for me	-0.72			
3) I take notice of the opinions of family and friends	-0.53			
Perceived behavioural control				
1) I am reluctant to be examined by a male doctor	0.35	2.08	11.56	0.68
2) I am uncomfortable asking what I want to know	0.64			
3) I do not find it easy to speak honestly about sexual matters	0.60			
4) I do not have time to visit the clinic for an examination	0.54			
5) I am worried about the cost of the examination	0.39			
6) I live far from clinics and health centers	0.42			
Behavioural intentions to undergo Pap testing				
1) I can undergo a Pap test even without a recommendation	-0.81	1.26	6.98	0.90
2) I can undergo a Pap test even without abnormal signs	-0.85			
3) I can undergo a Pap test according to my own will	-0.86			
4) I can undergo a Pap test regularly	-0.76			
KMO = 0.82; Bartlett’s test = 3100.83; *p* < 0.001; cumulative variance = 58.30; total Cronbach’s α = 0.77				
Stigma: If I get a gynaecological examination:				
1) People would avoid me	0.80	8.49	77.14	0.98
2) People would think I was unclean	0.88			
3) People would think badly of me	0.90			
4) People would not want to be friends with me	1.03			
5) People would be disgusted by me	0.98			
6) People would feel uncomfortable around me	0.87			
Shame: If I get a gynaecological examination:				
1) I would feel ashamed	0.93	0.93	8.48	0.94
2) I would feel embarrassed	0.89			
3) I would feel guilty	0.76			
4) I would feel scared	0.78			
5) I would feel disappointed in myself	0.69			
KMO = 0.94; Bartlett’s test = 7247.32; *p* < 0.001; Cumulative variance = 85.62; total Cronbach’s α = 0.97				

Pap, Papanicolau; HPV, human papilloma virus; NA; not applicable; KMO, Kaiser-Meyer-Olkin measure.

The 11 items of stigma and shame related to a gynaecological examination were assessed on four-point scales ranging from 1 (not at all) to 4 (very much). Higher scores indicated that the respondents perceived that greater degrees of stigma and shame were associated with the gynaecological examination. The results of the content validation phase revealed that two items out of 11 were rated differently (three different scores by the different experts). Nine items received the same score (i.e., a score of 4) from the three experts. Thus, all 18 items were used for construct validation.

The reliabilities of the stigma and shame scales were confirmed by Cronbach’s α values of 0.98 and 0.94, respectively (total Cronbach’s α = 0.97). Total Cronbach’s α values of 0.94 [14] and 0.95 [25] had been estimated for these variables in a previous study. A normal distribution was confirmed, the adequacies for factor analysis were measured, and the principal axis rotation extraction with oblimin rotation technique was used, as for the TPB variables. The values obtained for the KMO measure and Bartlett’s test of sphericity were 0.94 and χ^2^ = 7242.32 (*p* < 0.001), respectively. The stigma and shame factors accounted for 85.62% of the total variance, and explained 77.14% and 8.48% of the variance, respectively (Table 1).

### Statistical analysis

The data were analysed using descriptive analyses, the χ^2^ test for homogeneity of nominal variables, and independent *t*-tests for continuous variables. There were no missing values for any of the variables, because the online system was set up to require each participant to complete each item (except for the sociodemographic items).

The assumptions about normal distributions were confirmed prior to factor analysis and stepwise regression. Factor analysis was performed to identify the construct validity of the TPB variables, stigma, and shame.

Differences in measurement variables by level of sexual experience were analysed using the independent *t*-test. Associations between independent variables [awareness of Pap testing (after receiving information about the test), attitudes, norms, perceived control, stigma, and shame] and sociodemographic characteristics in relation to the dependent variable (intention to undergo a Pap test) according to the level of sexual experience were analysed using correlation analysis and the Pearson correlation coefficient.

Variables yielding significant associations at *p* < 0.05 were included in a stepwise multiple regression to build a model between the dependent variable (the intention to undergo a Pap test) and independent variables, by the level of sexual experience. The fitness of the regression model was examined using the *F*, *p*, *R*^2^, and adjusted *R*^2^ values. The multicollinearity test among independent variables was examined as tolerance and variance inflation factors, and the interdependence among residuals, was examined using the Durbin–Watson statistic. All analyses were performed using the SPSS Statistics 20 software (IBM, Armonk, NY, USA).

## Results

### Characteristics of the study sample

The mean age of the students was 20.47 ± 1.70 years (mean ± standard deviation, range 17–31 years). The data for five of the 480 subjects were incomplete, so the responses of these subjects were not included in the analysis. Of the remaining 475 subjects, 73.1% were not sexually experienced (*n* = 351) and 25.8% were sexually experienced (*n* = 124). The distribution of subjects among the eight classified administrative regions was as follows: Seoul (17.5%, *n* = 84), Gyung-gi (23.3%, *n* = 112), Chungcheong (10.4%, *n* = 50), Kangwon (11.9%, *n* = 57), Jeonbuk (10.2%, *n* = 49), Jeonnam (10.0%, *n* = 48), Kyungbuk (12.3%, *n* = 59), and Kyungnam (4.4%, *n* = 21). The study sample was not exactly representative of each region of Korea. However, 40.8% of the students in our sample were attending universities in Seoul and Gyung-gi, which was similar to the actual proportion of female university students attending in the same regions (i.e., 42%; personal communication, Korean Educational Development Institute [KEDI], August 14, 2013). The results for the distribution of degree majors indicated that 23.5% (*n* = 59) were medicine and nursing, 20.6% (*n* = 99) were business administration and human science, 19.8% (*n* = 95) were public health, 18.1% (*n* = 87) were engineering and science, and 12.5% (*n* = 60) were arts majors. Thus, there was even distribution of participants across majors.

### Awareness of the Pap test and cervical cancer prevention by sexual experience

The results for the opinions toward undergoing Pap testing indicated that 51.9% thought Pap testing is necessary before marriage and 40.3% did not know when Pap testing is necessary. There were no significant differences in this variable by level of sexual experience. Even after the students were given information about the importance of regular Pap testing, most of them perceived it to be less important (score, 77.76) than other cervical cancer prevention methods and were not confident that it is an effective method of cervical cancer prevention for themselves (score, 59.56).

Analysis of awareness of cervical cancer prevention methods revealed that there were also significant differences in awareness of the importance of abstinence from sexual intercourse until marriage (*t* = 41.77, *p* < 0.001) and regular use of condoms (*t* = 6.81, *p* = 0.009) between the two groups. Women who were sexually experienced were significantly less confident in regular Pap testing (*t* = 9.19, *p* = 0.003), abstinence from sexual intercourse until marriage (*t* = 72.74, *p* < 0.001), and regular condom use (*t* = 16.78, *p* < 0.001; Table 2) for the prevention of cervical cancer.

**Table 2:** **Awareness of the pap testing and cervical cancer prevention and socio demographic characteristics (*n*** **= 475, missing values excluded)**

**Characteristic**	**Category**	**Total**	**Not sexually experienced (*n*** **= 351)**	**Sexually experienced (*n*** **= 124)**	
			***n *** **(%) or mean ± SD**		***χ***^**2 **^**( *p *) or *t *** **( *p *)**
Opinions about the Pap test					
1) Pap testing is not necessary if I am HPV vaccinated		20 (4.3)	14 (4.1)	6 (4.9)	2.88 (0.410)
2) I don’t know when the pap test is necessary		187 (40.3)	144 (42.2)	43 (35.0)	
3) Pap testing is necessary after marriage		16 (3.4)	13 (3.8)	3 (2.4)	
4) Pap testing is necessary even before marriage		241 (51.9)	170 (49.9)	71 (57.7)	
Importance of cervical cancer prevention method (after giving the tip about the Pap test)					
(not at all, 0; very much, 100)					
–How important are the following methods for preventing cervical cancer?					
1) HPV vaccination		87.95 ± 16.73	87.89 ± 17.11	88.11 ± 15.65	0.15 (0.902)
2) Regular Pap testing		77.76 ± 22.00	78.45 ± 21.91	75.78 ± 22.26	1.29 (0.257)
3) Abstinence from sex until marriage		83.76 ± 21.99	87.51 ± 18.40	73.01 ± 27.36	41.77 (<0.001)
4) Minimizing the number of sexual partners		86.23 ± 19.78	87.22 ± 18.70	83.42 ± 22.41	3.27 (0.071)
5) Using condoms regularly		92.47 ± 15.80	93.60 ± 14.46	89.24 ± 18.81	6.81 (0.009)
Self-confidence of practices to prevent cervical cancer (after giving the tip about the Pap test)					
(not at all, 0; very much, 100)					
–How confident are you in the idea of practicing the following methods for preventing cervical cancer?					
1) HPV vaccination		76.46 ± 25.83	77.78 ± 24.30	72.76 ± 29.51	3.30 (0.070)
2) Regular Pap testing		59.56 ± 27.11	61.85 ± 26.64	53.09 ± 27.49	9.19 (0.003)
3) Abstinence from sex until marriage		80.80 ± 27.40	86.90 ± 21.14	63.64 ± 34.86	72.74 (<0.001)
4) Minimizing the number of sexual partners		85.35 ± 22.29	85.62 ± 21.71	84.59 ± 23.91	0.02 (0.670)
5) Using condoms regularly		86.98 ± 21.39	89.34 ± 19.96	80.37 ± 23.86	16.78 (<0.001)
Age	17–20 years	261 (55.3)	212 (60.9)	49 (39.5)	16.94 (<0.001)
	21–31 years	211 (44.7)	136 (39.1)	75 (60.5)	
		20.47 ± 1.70	20.19 ± 1.55	21.27 ± 1.89	39.22 (<0.001)
Religion	No	286 (61.4)	205 (59.6)	81 (66.4)	1.56 (0.19)
	Yes	180 (38.6)	139 (40.4)	41 (33.6)	
Monthly allowance	10,000 to <250,000	216 (48.4)	181 (55.0)	35 (29.9)	21.77 (<0.001)
(Korean won; 1,000 Korean won, 1 USD)	250,000 to 1,500,000	230 (51.6)	148 (45.0)	82 (70.1)	
		24.91 ± 15.05	22.05 ± 12.35	32.85 ± 18.93	48.72 (<0.001)
Heard of Pap test prior to survey	No	182 (38.6)	150 (43.1)	32 (25.8)	11.55 (0.001)
	Yes	290 (61.4)	198 (56.9)	92 (74.2)	
HPV vaccine	Not yet vaccinated	376 (83.4)	278 (84.2)	98 (81.0)	0.68 (0.411)
	Vaccinated	75 (16.6)	52 (15.8)	23 (19.0)	
Family history of	No	452 (96.4)	335 (96.0)	117 (97.5)	(0.59)*
Cervical cancer	Yes	17 (3.6)	14 (4.0)	3 (2.5)	
Age of first sexual			NA	19.69 (±1.50)	
Experience (years)					
Min–max: 13–23					
Condom use during	Never		NA	6 (5.7)	
Sexual intercourse	Occasionally		NA	32 (30.2)	
	Often		NA	37 (34.9)	
	Always		NA	31 (29.2)	

Pap, Papanicolau; HPV, human papilloma virus; *Fisher’s exact test.

Except where indicated otherwise, data are *n* (%) or mean ± SD values.

There were also significant differences in age (*χ*^2^ = 16.94, *p* < 0.001), monthly allowance (*χ*^2^ = 21.77, *p* < 0.001), and having heard of the Pap test (*χ*^2^ = 11.55, *p* = 0.001) between respondents who were sexually experienced and respondents who were not (Table 2).

### TPB variables (intention, attitudes, norms, and perceived control), stigma, and shame by sexual experience

There were no significant differences in the total scores of variables between the sexually experienced and inexperienced groups, but there were significant differences between some items of the TPB variables, and shame. The sexually experienced students took less notice of the opinions of family and friends (*t* = 6.56, *p* = 0.001) and had a less negative perception of an examination by a male doctor (*t* = 3.86, *p* = 0.05) (Table 3). They worried more about the cost of the examination (*t* = 8.48, *p* = 0.004), found it easier to ask what they wanted to know (*t* = 5.00, *p* = 0.026), felt more guilty at a gynaecological examination (*t* = 6.65, *p* = 0.010), and had a greater intention to undergo Pap testing even in the absence of abnormal signs (*t* = 5.01, *p* = 0.026; Table 3).

**Table 3:** Differences in Theory of Planned Behaviour variables related to Pap testing, and stigma and shame variables, by level of sexual experience

**Variable**		**Not sexually experienced (*n*** **= 351)**	**Sexually experienced (*n*** **= 124)**	
	**Items**	**Mean ± SD**		***t *** **( *p *)**
Behavioural attitudes				
1) Pap testing is helpful for early detection of cervical cancer		3.27 ± 0.55	3.38 ± 0.62	3.34 (0.068)
2) Pap testing is effective for curing cervical cancer		3.13 ± 0.53	3.17 ± 0.65	0.57 (0.452)
3) Pap testing is helpful for health management		3.20 ± 0.53	3.25 ± 0.62	0.68 (0.411)
4) Pap testing makes me confident about my health status		3.15 ± 0.55	3.17 ± 0.63	0.09 (0.766)
5) Pap testing is reliable for diagnosing cervical cancer		3.11 ± 0.55	3.19 ± 0.61	2.19 (0.140)
	Subtotal	15.86 ± 2.13	16.16 ± 2.60	1.65 (0.199)
Subjective norms				
1) Family and friends think Pap testing is necessary for detecting cervical cancer		3.09 ± 0.70	3.15 ± 0.75	0.68 (0.412)
2) Family and friends think Pap testing is necessary for detecting cervical cancer for me		2.56 ± 0.93	2.58 ± 0.84	0.05 (0.830)
3) I take notice of the opinions of family and friends		2.94 ± 0.67	2.76 ± 0.73	6.56 (0.011)
	Subtotal	8.59 ± 1.81	8.47 ± 1.84	0.40 (0.528)
Perceived behavioural control				
1) I am reluctant to be examined by a male doctor		3.32 ± 0.70	3.17 ± 0.81	3.86 (0.05)
2) I am uncomfortable asking what I want to know		2.67 ± 0.73	2.49 ± 0.88	5.00 (00.26)
3) I do not find it easy to speak honestly about sexual matters		2.76 ± 0.72	2.68 ± 0.85	0.96 (0.328)
4) I do not have time to visit a clinic for an examination		2.57 ± 0.76	2.51 ± 0.77	0.65 (0.419)
5) I am worried about the cost of the examination		3.04 ± 0.75	3.27 ± 0.71	8.48 (0.004)
6) I live far from clinics and health centers		2.35 ± 0.84	2.28 ± 0.82	0.56 (0.455)
	Subtotal	16.71 ± 2.80	16.40 ± 2.82	1.13 (0.288)
Behavioural intentions to undergo Pap testing				
1) I can undergo a Pap test even without a recommendation		2.59 ± 0.71	2.70 ± 0.70	2.11 (0.147)
2) I can undergo a Pap test even without abnormal signs		2.67 ± 0.68	2.83 ± 0.66	5.01 (0.026)
3) I can undergo a Pap test according to my own will		2.76 ± 0.65	2.89 ± 0.68	3.42 (0.065)
4) I can undergo a Pap test regularly		2.50 ± 0.70	2.58 ± 0.74	1.13 (0.288)
	Subtotal	10.57 ± 2.39	11.03 ± 2.45	3.18 (0.075)
Stigma: If I get gynaecological examination:				
1) People would avoid me		1.51 ± 0.58	1.45 ± 0.60	0.90 (0.344)
2) People would think I was unclean		1.48 ± 0.60	1.48 ± 0.60	0.02 (0.893)
3) People would think badly of me		1.49 ± 0.61	1.47 ± 0.60	0.12 (0.727)
4) People would not want to be friends with me		1.47 ± 0.60	1.40 ± 0.54	1.36 (0.243)
5) people would be disgusted by me		1.43 ± 0.59	1.41 ± 0.57	0.07 (0.794)
6) People would be uncomfortable around me		1.44 ± 0.61	1.41 ± 0.60	0.23 (0.631)
	Subtotal	8.82 ± 3.42	8.61 ± 3.26	0.34 (0.557)
Shame: If I get a gynaecological examination:				
1) I would feel ashamed		1.59 ± 0.71	1.72 ± 0.84	2.85 (0.091)
2) I would feel embarrassed		1.77 ± 0.84	1.85 ± 0.86	0.78 (0.379)
3) I would feel guilty		1.44 ± 0.62	1.62 ± 0.77	6.65 (0.010)
4) I would feel scared		1.44 ± 0.58	1.53 ± 0.67	2.21 (0.138)
5) I would feel disappointed in myself		1.46 ± 0.61	1.57 ± 0.70	2.64 (0.104)
	Subtotal	7.70 ± 3.01	8.29 ± 3.41	3.30 (0.070)

Scores range from “not at all” (1) to “very much” (4).

Pap, Papanicolau.

### Factors associated with the intention to undergo Pap testing by sexual experience

Tables 4 and 5 present the results for the associations between awareness of the importance of the Pap test, confidence in Pap testing, TPB variables, stigma, shame, and intention to undergo Pap testing by sexual experience. The results of the adjusted multiple regression analysis revealed that factors associated with the intention to undergo Pap testing in sexually inexperienced students were behavioural attitudes (B = 0.14, *p* = 0.021), subjective norms (B = 0.27, *p* < 0.001), and previously hearing about the Pap test (B = 0.13, *p* = 0.015). The corresponding factors in sexually experienced students were behavioural attitudes (B = 0.18, *p* = 0.03), subjective norms (B = 0.35, *p* < 0.001), perceived behavioural control (B = -0.25, *p* = 0.001), awareness of the importance of regular Pap testing (B = 0.16, *p* = 0.047), and confidence in a regular Pap test (B = 0.20, *p* = 0.02). The adjusted *R*^2^ scores of variance for sexually inexperienced and sexually experienced students were 14.9% and 42.9%, respectively.

**Table 4:** Factors associated with the intention to undergo pap testing by level of sexual experience

**Variables**	**Behavioural intention to undergo a Pap test**	
	**Not sexually experienced (*n*** **= 351)**	**Sexually experienced (*n*** **= 124)**
	***r *** **( *p *)**	***r *** **( *p *)**
Behavioural attitudes* (scores; 5–20)	0.29 (<0.001)	0.45 (<0.001)
Subjective norms** (scores; 3–12)	0.34 (<0.001)	0.48 (<0.001)
Perceived behavioural control*** (scores; 6–24)	-0.008 (0.891)	-0.27 (0.004)
Stigma**** (scores; 6–24)	-0.04 (0.471)	-0.31 (0.001)
Shame****** (scores; 5–20)	-0.18 (0.001)	-0.27 (0.014)
Socio demographic characteristics		
Age	-0.06 (0.310)	0.006 (0.950)
Religion (yes/ no)	-0.13 (0.023)	-0.14 (0.132)
Monthly allowance	-0.02 (0.711)	0.12 (0.215)
Heard of Pap test prior to survey (yes/no)	0.14 (0.011)	0.014 (0.880)
HPV vaccination (yes/no)	-0.001 (0.986)	-0.04 (0.679)
Family history (yes/no)	0.008 (0.881)	-0.16 (0.091)
Regular condom use	NA	0.06 (0.564)
Importance of regular Pap testing (scores; 0–100)	0.17 (0.002)	0.32 (<0.001)
Confidence about regular Pap testing (scores; 0–100)	0.23 (<0.001)	0.49 (<0.001)

Pap, Papanicolau, HPV, human papilloma virus, NA; not applicable.

*Higher scores indicate higher perception of benefit from Pap test,

**Higher scores indicate more likely to listen to others.

***Higher scores indicate greater perception of barriers to undergoing the Pap test.

****Higher scores indicate more likely to feel stigma associated with gynaecological examination.

******Higher scores indicate more likely to feel shame about gynaecological examination.

**Table 5:** Factors influencing the intention to undergo Pap testing, by level of sexual experience

**Variable**	**Not sexually experienced (*n*** **= 351)**								**Sexually experienced (*n*** **= 124)**							
	**Non-standardized coefficients**		**Standardized coefficients**	**Adjusted *R***^**2**^	***t***	***p***	**Tolerance**	**VIF**	**Non-standardized coefficients**		**Standardized coefficients**	**Adjusted *R***^**2**^	***t***	***P***	**Tolerance**	**VIF**
	**Beta**	**Standard error**	**Beta**						**Beta**	**Standard error**	**Beta**					
Constant	4.03	1.01	-	-	3.97	<0.001			5.61	1.60	-	-	3.50	0.01		
Behavioural attitudes* (scores; 5–20)	0.16	0.07	0.14	1.2	2.32	0.021	0.747	1.338	0.17	0.08	0.18	3.2	2.17	0.03	0.779	1.284
Subjective norms ** (scores; 3–12)	0.35	0.08	0.27	11.9	4.49	<0.001	0.757	1.322	0.46	0.11	0.35	23.8	4.31	<0.001	0.769	1.300
Perceived behavioural control*** (scores; 6–24)									-0.22	0.06	-0.25	5.4	-3.40	0.001	0.945	1.058
Heard of Pap test prior to survey (yes/no)	0.61	0.26	0.13	1.8	2.43	0.015	0.983	1.017								
Importance of regular Pap testing (scores; 0–100)									0.02	0.01	0.16	1.6	2.01	0.047	0.774	1.292
Confidence in regular Pap testing (scores; 0–100)									0.18	0.01	0.20	8.9	2.36	0.02	0.719	1.392
*F* = 8.99 (*p* < 0001); cumulative adjusted *R*^2^ = 14.9									*F* = 17.68 (*p* < 0.001); cumulative adjusted *R*^2^ = 42.9							

Pap, Papanicolau ; VIF, variance inflation factors.

*Higher scores indicate higher perception of benefit from Pap test,

**Higher scores indicate more likely to listen to others.

***Higher scores indicate greater perception of barriers to undergoing the Pap test.

## Discussion

In general, the results indicated that there was low awareness of the importance of the Pap test and low confidence in Pap testing as a means of preventing cervical cancer in this population. Approximately one third (38.6%) of the students had not heard of the Pap test, and 40.3% had no opinion on about the test prior to this survey. These results indicate that there is a need for the provision of basic information regarding Pap testing and its role in cervical cancer prevention to university students, regardless of their level of sexual experience.

The number of new cases of cervical cancer in Korea has been decreasing, but the total number of women being treated and the treatment costs are constantly increasing. During 2006–2010 there was a 6.5% increase in the rate of cervical cancer diagnoses in women younger than 19 years, and this age group had the highest rate of increase of all women aged 19–69 years during this time period [8]. The scores for the intention to undergo Pap testing in this study ranged from 10.5 to 11.0 out of 16, which was lower than the scores previously reported for Western university students (93.8%) [7]. This result could be a consequence of cultural differences. In the present study, 25.8% of the students reported that they had experienced sex. A previous study of Korean female students found that 19.0–41.6% of them had experienced sexual intercourse [6,26,27]. The differences between these studies could be attributable to differences in the sample size, the geographical location of the survey, and the frankness of the respondents. However, there is a clear increasing trend toward sexual activity among university students in Korea. Health professionals should be concerned about the active prevention of cervical cancer in this population. The population of unmarried women who are sexually experienced will likely increase, because the average age at first marriage of Korean women is increasing, and was 29.2 years in 2012 [28].

The students in this study lacked awareness of the importance of regular Pap testing and had little confidence in its effectiveness as a method of cervical cancer prevention. Approximately one half (51.9%) of the students responded that Pap testing is necessary before marriage. However, Pap screening was rated lowest among the five methodologies of cervical cancer prevention, with respect to the perception of importance and confidence in its effectiveness, regardless of the level of sexual experience. The age at which a women first learns of Pap testing has been reported as the most important determinant of the intention to undergo the test [7]. Taken together, these findings suggest that education about Pap testing should be provided to young women as early as possible. Female university students should also be informed of the importance of Pap testing, and health professionals should encourage students to initiate and to continue regular Pap screening if they are sexually active.

Awareness and confidence regarding condom use was highest among the five methods of cervical cancer prevention, regardless of the students’ level of sexual experience. This result suggests that condom use is well-accepted as a method to protect of sexual health in the university setting. This finding agrees with a previous finding that 72.2% of sexually experienced university students report using condoms for contraception [26]. Condom use should be reinforced; although this type of prophylaxis does not fully prevent HPV infection, it may have a protective effect against genital warts, dysplasia, and invasive cervical cancer [29]. The prevalence of HPV infection among Korean female university students was 15.2% in 2004, which was similar to the prevalence in Western countries [9]. However, in 2011, the prevalence of an intention to request the HPV vaccine was 25.0% [30]. It is noteworthy that the students in the present study were less confident regarding the HPV vaccination despite being well aware of its importance for cervical cancer prevention. This difference might be the high cost of HPV vaccination, and the lack of financial assistance from the government and private insurance companies. As mentioned earlier, HPV vaccination is not mandatory in Korea [6]. Therefore, each student must pay the full cost of the vaccination, which can be as high as the equivalent of 400 USD.

In addition to encouraging young women to undergo Pap screening to prevent cervical cancer, health professionals and administrators in Korea must now develop a strategy for implementing a nationwide HPV vaccination program. Moreover, previous research findings have consistently indicated that there is a lack of awareness about HPV, and that young women express relatively optimistic attitudes about HPV infection [5,30,31]. Therefore, education about methods of HPV prevention (including HPV vaccination) should be organised and implemented throughout the Korean university system.

Compared with the students who were not sexually experienced, the students who were sexually experienced responded that condom use was less important for cervical cancer prevention, and they were also less confident about undergoing the Pap test. This result suggests that sexually experienced students were not more aware of, or confident in, methods to prevent cervical cancer. Therefore, there is an urgent need to inform university students about methods of cervical cancer prevention. Information about the importance of condom use and the Pap test should be especially targeted at sexually experienced students.

A particularly notable finding of this study was that the subjective norm (one of the TPB attitudes) was the most important predictor of a woman’s intention to undergo Pap testing, regardless of her level of sexual experience. This result is consistent with a previous study finding that young women are more likely to learn about Pap testing from their friends and family than from their health-care providers. These women are likely receiving inaccurate information about Pap testing [7]. This finding highlights the need to deliver accurate information to the general population and community health professionals.

While there were no significant differences in the total scores for the TPB variables, or for stigma and shame by level of sexual experience, there were some significant differences for the individual items. Based on these results, the following strategies to increase the intention to undergo Pap screening should be introduced, and should be adapted to the level of sexual experience. Students who are not sexually experienced should be educated repeatedly about the benefits of Pap testing. Educational interventions aimed at students who are already sexually experienced should focus on improving their positive attitudes about the Pap test, their confidence in the Pap screening program, and on minimising perceived barriers to Pap screening.

The results for social approval for Pap screening in both groups indicate that the roles of the family and friends should be also addressed. The bivariate analysis revealed that students who were not sexually experienced were more likely to listen to the opinions of their family and friends, were more concerned about receiving an examination from a male doctor, were less comfortable asking questions about the procedure, and were less likely to request a Pap test when no abnormal signs were present. To address these issues, universities could provide information about cervical cancer prevention on their website homepages that includes the gender of each professional that performs Pap testing in the various community clinics. Information about Pap screening guidelines after first-time sexual intercourse, Pap procedures (interval and preparation), importance of regular testing without signs or symptoms, and “questions and answers” forum for their students could also be provided.

Pap test education for university campus students should be expanded and the importance of the Pap test for young women should be conveyed to the general community population. The result that sexually experienced students were more concerned about the cost of the examination suggests that universities could inform students (e.g., using media and online sites) about the locations of clinics and health centres that offer free or low cost Pap testing.

The feelings of guilt expressed by sexually experienced students in relation to visiting a gynaecological clinic should be carefully managed to reduce these negative feelings. Emotional reactions such as stigma and shame interfere with students’ intentions toward health behaviours [14,25,32]. In the present study, stigma and shame were not significant predictors of intention, but they were significantly related to intention in the bivariate analyses. The students participating in this study had never actually experienced a Pap test. Most of them were presumably only imagining the scene of a gynaecological examination, so this study was not able to measure the real emotional impact from the experience of a gynaecological examination. Stigma and shame should be mediated to increase the intention to undergo a Pap test. Shameful feelings should be managed and reduced, particularly in sexually experienced students. Female university students should be empowered by the knowledge that visiting clinics or health centres for Pap screening is very natural and critically important. The prejudice and other negative attitudes toward gynaecological examination among the general public should be corrected. It is important that the general population understands that Pap screening is necessary regardless of marital status, and that young women have a right to visit a gynaecological clinic for the early detection of cervical cancer.

One limitation of this study was that random sampling of the population was not applied. There are 341 universities in Korea, and an estimated 451,080 female students attended these universities during 2012–2013 (personal communication, KEDI, August 14, 2013). Geographical distributions and the ratios of students in the Seoul and Gyung-gi areas were considered for selecting students. Respondents were not actually followed-up regarding their Pap testing. Their responses were hypothetical because this study focused only on intentions of students who had never experienced a Pap test. The regression coefficients for intention were not high (range 14.9–42.9%), particularly among the students who were not sexually experienced. This result suggests that additional hypotheses should be considered in future studies. Sociodemographic factors such as smoking and consumption of alcohol are associated with the intention to prevent cervical cancer [15], but these factors were not examined in the present study. The positive effect of religion on intention to undergo Pap testing was only significant among the students who were not sexually experienced. The roles of important sociodemographic factors in unmarried women who have actually undergone Pap testing should be clarified in future studies.

This study is the first descriptive survey to measure the awareness of, the intention to undergo, and the influencing factors of intention towards, Pap screening in unmarried university students in Korea. The use of an online survey likely encouraged students to honestly express their opinions and attitudes about their sexual experience. Despite the use of convenience sampling, the subject population reflected the geographic diversity of the university system. The study results represent baseline data for university students in Korea.

## Conclusions

The findings of this study indicated that, in general, there was low awareness of the Pap test among unmarried university women. Level of sexual experience was helpful for understanding the differences in awareness of the Pap test, and the factors associated with the intentions to undergo a Pap test among the students. Strategies to increase the intention to undergo Pap screening should be introduced, and should be adapted to the level of sexual experience.

Health professionals in Korea should be responsible for the provision of education about Pap testing, especially for young or unmarried women. The awareness of the importance of Pap screening should be increased among the general population.

## Abbreviations

Pap: Papanicolau; HPV: Human papilloma virus; TPB: Theory of planned behaviour; KEDI: Korean educational development Institute; KMO: Kaiser–Meyer–Olkin.

## Competing interests

The author declares that she has no competing interests.

## Authors’ contributions

HWK was entirely responsible for acquiring the funding for the study, for performing all stages of the research, and for writing the manuscript.

## Pre-publication history

The pre-publication history for this paper can be accessed here:

http://www.biomedcentral.com/1472-6874/14/100/prepub
